# The significance of droughts for hyporheic dwellers: evidence from freshwater crayfish

**DOI:** 10.1038/srep26569

**Published:** 2016-05-26

**Authors:** Antonín Kouba, Jan Tíkal, Petr Císař, Lukáš Veselý, Martin Fořt, Josef Příborský, Jiří Patoka, Miloš Buřič

**Affiliations:** 1 University of South Bohemia in České Budějovice, Faculty of Fisheries and Protection of Waters, South Bohemian Research Center of Aquaculture and Biodiversity of Hydrocenoses, Zátiší 728/II, CZ-389 25 Vodňany, Czech Republic; 2 Department of Zoology and Fisheries, Faculty of Agrobiology, Food and Natural Resources, Czech University of Life Sciences Prague, Kamýcká 129, CZ-165 21 Prague 6-Suchdol, Czech Republic

## Abstract

Freshwater biodiversity is globally threatened by various factors while severe weather events like long-term droughts may be substantially devastating. In order to remain in contact with the water or stay in a sufficiently humid environment at drying localities, the ability to withstand desiccation by dwelling in the hyporheic zone, particularly through vertical burrowing is crucial. We assessed the ability of three European native and five non-native crayfish as models to survive and construct vertical burrows in a humid sandy-clayey substrate under a simulated one-week drought. Three native species (*Astacus astacus*, *A. leptodactylus*, and *Austropotamobius torrentium*) suffered extensive mortalities. Survival of non-native species was substantially higher while all specimens of *Cherax destructor* and *Procambarus clarkii* survived. The native species and *Pacifastacus leniusculus* exhibited no ability to construct vertical burrows. *Procambarus fallax* f. *virginalis* and *P. clarkii* constructed bigger and deeper burrows than *C. destructor* and *Orconectes limosus.* In the context of predicted weather fluctuations, the ability to withstand desiccation through constructing vertical burrows into the hyporheic zone under drought conditions might play a significant role in the success of particular crayfish species, as well as a wide range of further hyporheic-dwelling aquatic organisms in general.

Freshwater ecosystems occupy less than 1% of the Earth’s surface, but support approximately one tenth of the world’s species and one third of all vertebrates^1^. These systems provide a wide range of valuable services also to human populations. The increased demand on freshwater resources has led to a freshwater crisis in both human and biodiversity perspectives^2^. While the conservation status and distribution of freshwater taxa is less well-known than that of terrestrial species^3^, there is growing evidence that freshwater taxa are at greater risk of extinction than those in terrestrial or marine ecosystems^4^,^5^,^6^, making freshwater conservation a priority^7^.

Freshwater crayfish (Crustacea, Decapoda, Astacida) are considered not only keystone species in freshwaters but also strong ecosystem engineers modifying the environment to suit themselves. Indigenous crayfish species (ICS) have been often designated as e.g., bioindicator, umbrella, or even flagship species in aquatic conservation^7^,^8^. Three prominent crayfish invaders of North American origin (spiny-cheek crayfish *Orconectes limosus*, signal crayfish *Pacifastacus leniusculus*, and red swamp crayfish *Procambarus clarkii*) have been introduced to Europe between 1890 and the mid-1970s and became particularly widespread across the continent^9^. Thousands of populations of native European astacids have been lost, and many more have been substantially reduced, largely due to direct or indirect effects of the presence of non-indigenous crayfish species (NICS). These not only influence their European counterparts by competition but especially spread the causative pathogen of crayfish plague (an oomycete *Aphanomyces astaci*), causing mass mortalities to crayfish not originating from North America. Apart from imposing strong competitive pressures on native crayfish populations, these invaders possess the ability to alter food webs and entire ecosystems^10^. The main reason for NICS introductions in Europe was initially their expected commercial use (fisheries and aquaculture)^11^. In recent years, however, introductions of further NICS have usually involved escapes or intentional releases of aquarium-bred specimens^12^,^13^, making the situation more inauspicious.

Besides the expectation of high extinction rates in crayfish in general^7^,^14^, interactions with non-native crayfish are the leading cause of decline of the native counterparts^15^,^16^. Explanation of these displacements have been generally based on the evaluation of one, or a combination, of four biotic mechanisms: competition, predation, reproductive interference and disease transmission^15^, as well as lower environmental requirements in non-native crayfish^11^. However, an array of factors threatening biodiversity and aquatic ecosystems in general is much wider and more complex, including climate change and severe weather events like long-term droughts, unexpected floods, fires, heavy storms etc.^7^,^17^ having far reaching consequences^18^,^19^. Indeed, the role of abiotic disturbances such as long-term droughts on aquatic biota has remained overlooked and poorly understood for a long time^19^,^20^,^21^, and such knowledge is still scarce and fragmentary^22^,^23^,^24^. In the context of ongoing and predicted weather fluctuations^25^,^26^, the ability to withstand desiccation and particularly to be involved in vertical burrowing under severe drought conditions might play a significant role in the success of various crayfish. Similar kinds of adaptations have been documented for unionid mussels, gill-breathing snails^27^,^28^ and a wide range of aquatic insects^19^,^21^.

In this study, we assessed the ability of three European ICS and five NICS as models to survive simulated drought conditions and to construct vertical burrows in a humid sandy-clayey substrate as a protection against drought conditions.

## Results

**Figure 1:**
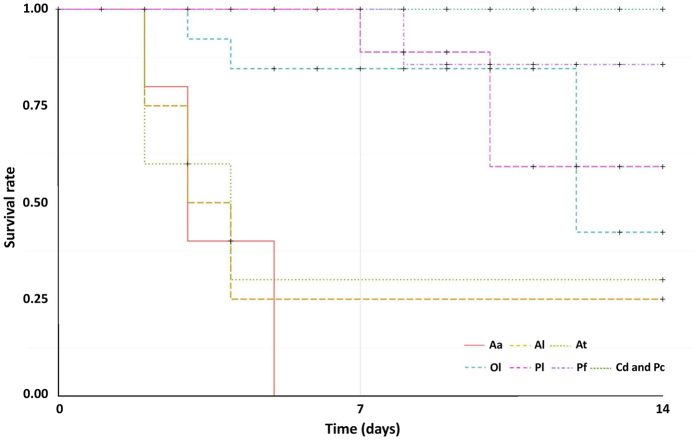
Kaplan-Meier survival analyses of crayfish species involved in the experiment. Legend refers to the particular species as follows: Aa–noble crayfish *Astacus astacus*, Al–narrow-clawed crayfih *Astacus leptodactylus*, At–stone crayfish *Austropotamobius torrentium*, Ol–spiny-cheek crayfish *Orconectes limosus*, Pl–signal crayfish *Pacifastacus leniusculus*, Pf–marbled crayfish *Procambarus fallax* f. *virginalis*, Cd–yabby *Cherax destructor*, and Pc–red swamp crayfish *Procambarus clarkii*.

Substantial differences were detected by means of survival analysis among studied species (χ^2^ = 44.3, df = 7, p ≤ 10^−6^). All specimens of red swamp crayfish, yabby and marbled crayfish survived the simulated one-week drought but certain post-treatment mortality was modeled for marbled crayfish during a one-week observation in aquaria with a final survival of 85.7 ± 13.2% (mean ± SD; Fig. 1). Relatively high survival rates of spiny-cheek crayfish and signal crayfish at the end of the simulated one-week drought (84.6 ± 10.0 and 88.9 ± 10.5%, respectively) were followed by post-treatment mortality resulting in final values of 42.3 ± 21.7 and 59.3 ± 18.5%, respectively. Narrow-clawed crayfish (*Astacus leptodactylus*) and stone crayfish (*Austropotamobius torrentium*) suffered substantial losses during simulated drought (25.0 ± 21.7 and 30.0 ± 23.9% survival, respectively) but the values remained stable thereafter. All noble crayfish (*Astacus astacus*) died within 5 days of simulated drought.

**Figure 2:**
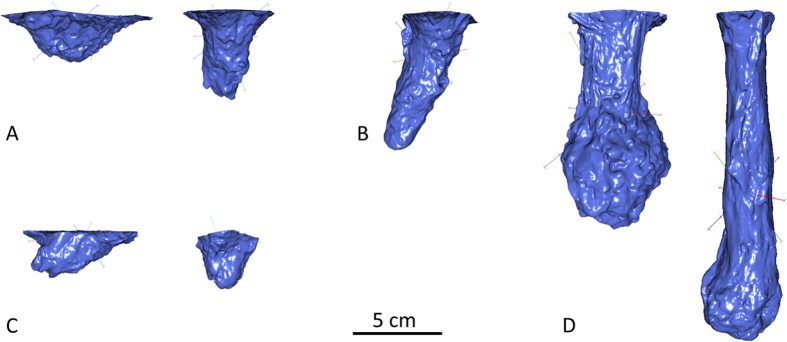
Examples of constructed burrows in yabby (**A**), marbled crayfish (**B**), spiny-cheek crayfish (**C**), and red swamp crayfish (**D**). 3D models of burrows of males (if present) are located on the left side of respective species. Further examples of burrows are available in supplementary materials (Fig. S1).

No attempts at vertical burrowing were observed in ICS (noble, stone and narrow-clawed crayfish) and signal crayfish. The remaining species exhibited different degrees of burrowing activity (Fig. 2). Crayfish usually constructed a single burrow in the suggested position (“initial burrow”). One red swamp crayfish and one yabby created two burrows and two other red swamp crayfish, both males, even dug three burrows in different places. The most prominent burrow was always located in the originally suggested depression. Only red swamp crayfish exhibited the ability to close the burrow entrance by means of a mud plug in our experiment. One out of twelve males created the plug but females were more active (n = 7) in doing this (Z = −2.050, p = 0.040).

**Figure 3:**
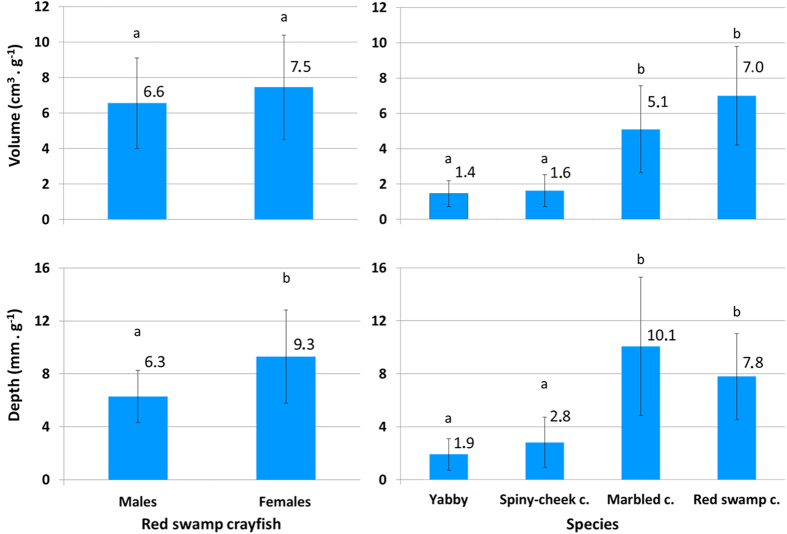
Relative volume and depth of burrows in burrowing crayfish species involved in the experiment. Significant differences between the sexes were detected only in case of red swamp crayfish–left column. Interspecific values are shown in the right column. Data are presented as mean ± SD. Values with differing letters within each graph are significantly different (P < 0.05).

Differences in burrowing between the sexes of particular species were detected only in red swamp crayfish (Fig. 2). Although the relative volume was comparable (t_24, 22_ = −0.670, p = 0.491), females constructed deeper burrows than males (t_24, 22_ = −2.989, p = 0.007). Marbled and red swamp crayfish constructed bigger (p < 0.020 and p < 10^−4^, respectively) and deeper burrows (p < 0.002 and p < 0.004, respectively) than did yabby and spiny-cheek crayfish (Fig. 3).

## Discussion

We conducted the first comparative study evaluating burrowing activity under drought conditions in both selected native and non-native crayfish species currently present in Europe. Taking the results as a whole, it should be understood that the outlined crayfish desiccation capacities and burrowing abilities possess a degree of simplification and are related to the experimental set up, thus crayfish responses might vary under specific conditions. For instance, crayfish facing desiccation at localities might be exposed to even worse conditions, e.g. in terms of higher temperature and lower air humidity^29^,^30^, the substrate might not be plastic enough for stability of burrows or the presence of coarse particles might prevent burrowing as such, i.e. substrate composition matters^24^,^31^. Also desiccation capacities and burrowing abilities of small-bodied juvenile animals are expectedly lower compared to sub-adults and young adults. Smaller animals have less water reserves compared to their relatively big body surface, allowing their desiccation; their physical ability to manipulate relatively big substrate particles is lower^29^,^30^. On the contrary, burrowing capacities of large-bodied specimens are likely also low due to difficulties with movement out of water (among others, the presence of big claws). Nevertheless, we believe it is unexpected that the patterns of desiccation capacities and burrowing abilities presented would change substantially among species under specific conditions. Also, the terminal stages of drought events when free water becomes unavailable at the localities are similar at both lotic and lentic sites, thus some degree of generalization is warranted.

Elevated desiccation capacities under different conditions have been documented among a wide range of NICS^29^,^32^,^33^. Our results clearly document substantially reduced survival of European ICS compared to NICS under simulated drought conditions, with absolute resistance in red swamp crayfish and yabby (Fig. 1), both considered to be warm-water species well adapted to conditions even in semiarid and arid regions^34^,^35^. Marbled crayfish suffered only post-treatment mortality with a final modeled survival of above 80%. The closely related slough crayfish *P. fallax* was found to cope less successfully with drought conditions than the Everglades crayfish *P. alleni*^24^,^36^; the latter has also been found accidentally released into European waters, although its establishment is considered unlikely^37^. High survival was also achieved by signal crayfish and spiny-cheek crayfish after the simulated drought followed by some post-treatment mortality (Fig. 1).

Resistance to desiccation is a necessary prerequisite for burrowing that mediates successful survival during severe droughts. A certain degree of burrowing is a habit present among crayfish. Less burrowing species just create short, unbranched burrows (or depressions) in the substratum, under stones, logs etc. They may also excavate burrows in the sides of clay banks^38^,^39^,^40^. Nevertheless, such burrowing activity might not be as adequate for survival as vertical burrowing under severe long-term droughts. European ICS species and signal crayfish exhibited no ability to construct vertical burrows. On the other hand, red swamp crayfish and marbled crayfish constructed bigger and deeper burrows than yabby and spiny-cheek crayfish (Fig. 3).

Considering their desiccation resistance and burrowing abilities, the red swamp crayfish is the most tolerant species we compared. It is worth mentioning that red swamp crayfish is also the only species in our experiment exhibiting the closing of the burrow entrance with a mud plug^31^,^41^, particularly in females, which also created deeper burrows than males (Fig. 2). Females frequently use burrows for egg incubation. Females with eggs are not usually submerged in the ground water due to low available levels of dissolved oxygen, and oxygen diffuses directly from the burrow atmosphere while egg are fanned by swimmeret movements^42^. However, deeper burrows constructed by females in our experiment suggest the possible importance of having better access to the water. Even signal crayfish and white-clawed crayfish *Austropotamobius pallipes* eggs artificially stored in a humid environment require incubation in aquatic conditions at least during final stages of embryonic development, likely due to increased metabolic waste excretion^43^, thus, at least periodical egg submergence can be expected in crayfish.

Besides red swamp crayfish, further NICS involved in the study exhibit combined strategies focusing on increased desiccation capacity (yabby, marbled crayfish and signal crayfish) and burrowing (marbled crayfish and to a lesser extent also yabby and spiny-cheek crayfish). The lowest success belongs to ICS in particular and signal crayfish in terms of burrowing. We consider desiccation capacity and burrowing as further, still largely overlooked factors^7^,^11^,^17^, whose importance will rise with ongoing and predicted weather fluctuations in the future^25^,^26^. Descriptions of current status and projections of droughts in European freshwater habitats together with current and future distributions of crayfish are beyond the scope of this article, however, the first suggestion might be that such events will be particularly pronounced among ICS in the warmer (Mediterranean) regions of the continent. Nevertheless, local extremes cannot be neglected and their importance will likely rise in the future too. For instance, a long-lasting drought hit Europe in 2015. It particularly affected Central and Eastern Europe while in some regions it was the driest (North Slovakia) and in others (Czech Republic and Poland) it was the second driest summer of the last 50 years–following 2003^44^. Expanding from its importance during droughts, burrowing also plays a role in overwintering which might increase the probability of establishment of non-indigenous species^45^,^46^. Following crayfish as a model group of freshwater organisms, a similar mode of action can be expected in further hyporheic-dwelling aquatic biota e.g. unionid mussels and clams, and a wide range of aquatic insects, as well as crabs and fish.

## Methods

### Container preparation

To create a suitable test substrate, sixteen kilograms of sand (České štěrkopísky Inc., Čavyně, Czech Republic) with a humidity of 5.2% and 24 kg of WBT clay (Keraclay, Plc., Brník, Czech Republic) with a humidity of 7.1% were thoroughly mixed by hand (=60% clay proportion expressed on a wet weight basis). For size distribution of sand and clay particles see Table S1. Aged tap water was added to get a final humidity of 16.5%. Our preliminary experiment revealed that the clay itself and a mixture with 80% clay proportion are too plastic to facilitate manipulation by crayfish. On the other hand, a substrate with 40% clay proportion was not stable enough for burrowing, which confirms the importance of substrate composition for successful burrowing^24^,^31^. The resultant humid mixture was used to fill a series of plastic containers (inner diameter = 34.0 cm, height = 44.5 cm) to a depth of ca. 34 cm. To better simulate natural conditions when certain areas with residual water persist at the drying-up localities^47^, a shallow “initial burrow” (diameter 2.6 cm, depth 1.3 cm; volume 6.9 cm^3^) was created in the margin of the container and 5 mL of water was added to stimulate burrowing in the suggested position. A single crayfish individual (see respective species and numbers below) was placed in the container. Each container was covered by a 0.5 cm thick polystyrene lid in order to prevent acute desiccation of the experimental animal. The air relative humidity (RH) reached at least 99% within an hour after coverage. The coverage was implemented in order to enhance survival of susceptible indigenous crayfish species allowing supposed burrowing. Natural conditions necessarily possess lower air humidity but there is a certain time period before free water becomes unavailable. It opens a space for animals to prepare a burrow, find water pools etc. For comparison, highly tolerant red swamp crayfish exposed to room air of approximately 50% RH showed mortality after 3–7 days’ exposure^30^ and all animals died at 30% RH and 24 °C within about a day^29^. The experimental temperatures (mean ± SD) of air and the sandy-clayey mixture were 20.2 ± 0.3 and 20.3 ± 0.1 °C, respectively. Temperature was registered hourly using Minikin loggers (Environmental Measuring Systems, Brno, Czech Republic). Fairly similar temperatures are often used in laboratory experiments and are relatively high—representing warm periods of the year when the most pronounced droughts usually occur. Although the temperatures might be even higher during such events^29^,^30^, we considered values close to 20 °C the best compromise, taking the requirements of the most sensitive species involved (the stone crayfish) into account^48^,^49^.

### Experimental animals

We selected intact (with all walking legs including well developed chelae) intermoult specimens of three European ICS and five NICS. These were usually adults based on biometry and secondary sexual characters, but a few subadults might also have been involved. The sex ratio was balanced except for marbled crayfish *Procambarus fallax* f. *virginalis* where only females occur. For reasons of conservation and following a lack of vertical burrowing activities (see results above), only limited numbers of ICS (n = 4 for each species) were used. Noble crayfish *Astacus astacus* were caught from the pond U Sudu (Těšínov u Protivína, Czech Republic; 49° 20′ N, 14° 28′ E) under permit no. KUJCK 4820/2011 OZZL/4/Ou, Regional Office of South Bohemian Region. Narrow-clawed crayfish *A. leptodactylus* were obtained from the limestone quarry Kosov (Jarov u Berouna, Czech Republic, 49° 56′ N, 14° 3′ E) under permit no. 123564/2012/KUSK, Regional Office of Central Bohemia Region, and stone crayfish *Austropotamobius torrentium* came from Zubřina brook (Havlovice, Czech Republic; 49° 12′ N, 14° 17′ E) based on permit no. ŽP/2450/2011, Regional Office of Plzeň Region. Both signal crayfish *Pacifastacus leniusculus* and spiny-cheek crayfish *Orconectes limosus* (n = 10 for both species) were caught from the wild populations in the Vysočina Region and from the Lipno Reservoir, South Bohemian Region, respectively. Marbled crayfish (n = 12), yabby *Cherax destructor* (n = 14), and red swamp crayfish *P. clarkii* (n = 24) were obtained from laboratory cultures. Considering the categorization by Hobbs^50^, all crayfish species involved in our experiment belong to the tertiary burrower category. Some members of this group are often incorrectly referred to as non-burrowers^39^ but they may respond to habitat drying by excavating shallow simple burrows into the hyporheic zone, although experiencing population declines and local extinctions during severe droughts^22^,^51^,^52^,^53^,^54^.

Crayfish were individually acclimatized for three days in a bucket with 8 L of aerated tap water, without feeding. Animal wet weights (to the nearest 0.1 g) and carapace lengths (to the nearest 0.1 mm) were determined and crayfish were placed in the experimental container for a one week period simulating drought conditions (for crayfish biometry see Table S2). Crayfish survival was evaluated daily. After one week’s exposure, surviving animals were collected and transferred to aquaria with water for one week to evaluate post-treatment mortality.

All experimental manipulations were conducted according to the principles of the Institutional Animal Care and Use Committee (IACUC) of the University of South Bohemia, Faculty of Fisheries and Protection of Waters, Research Institute of Fish Culture and Hydrobiology, Vodňany, based on the EU harmonized animal welfare act of Czech Republic. Nevertheless, no specific permissions were required for the locations and activities considering taxa involved in this study.

### Creation and measurement of casts

Gypsum casts of any burrows excavated were created after removal of animals. If direct collection of crayfish from burrows was impossible, a small amount of carbonated water was added to the burrow in order to evict animals, which led to success in most cases. If collection of animals was prevented (as occurred only with several specimens of red swamp crayfish), a new independent replication was conducted. Any excess water was removed from the burrow bottom by blotting with absorbent paper. Depth of casts was measured by a digital caliper to the nearest mm. Casts were further scanned by an Artec Spider™ hand-held 3D laser scanner (Artec Group, Luxenbourg) located at the Department of Cybernetics, Faculty of Applied Sciences, University of West Bohemia in Pilsen with a stated resolution of 0.1 mm and accuracy up to 0.03 mm. The scanner is based on the structured light principle and provides a 3D mesh of the object as an output, generated in real world coordinates (mm). The resulting STL (STereoLithography) mesh was imported to the Artec Studio, version 10 (Artec Group, Luxembourg) where the volume of the 3D mesh was calculated. As size naturally varies within and among crayfish species, relative data reflecting weight of respective animals were used in presentation of burrowing activity (volume and depth).

### Statistical analysis

Non-parametric Kaplan-Meier survival analyses were performed in the R-statistics software (version 3.2.4, R Development Core Team 2015), with the packages: “KMsurv” and “survival”. In addition, for graphical visualisation the packages “GGally” and “ggplot2” were employed. In assessing sex differences, the ability to close the burrow entrance by means of a mud plug was assessed as 1 or 0. Due to lack of normality and homoscedasticity of this dataset (evaluated with Kolmogorov–Smirnov and Levene’s tests, respectively; these tests uniformly used further if appropriate for testing assumptions of parametric tests), a non-parametric Mann-Whitney U was applied. Intersex differences in term of relative burrow depth and volume were compared with Student’s t-test. Because of heteroscedasticity in data, the non-parametric Kruskal-Wallis test followed by multiple comparisons of mean ranks for all groups was applied for interspecific comparisons (values of both sexes were pooled among species for this purpose). These data were analyzed using Statistica 12.0 (StatSoft, Inc.). The null hypothesis was rejected at α = 0.05 in all tests of this study.

## Additional Information

**How to cite this article**: Kouba, A. *et al.* The significance of droughts for hyporheic dwellers: evidence from freshwater crayfish. *Sci. Rep.* **6**, 26569; doi: 10.1038/srep26569 (2016).

## Supplementary Material

Supplementary Information
