# Supplementary material for: The significance of droughts for hyporheic dwellers: evidence from freshwater crayfish
Source: Sci Rep. 2016 May 26;6:26569. doi: 10.1038/srep26569 (PMC4880899; doi:10.1038/srep26569)
Supplement: Supplementary Information [file srep26569-s1.pdf]

Supplementary Information

The significance of droughts for hyporheic dwellers: evidence from freshwater crayfish

Antonín Kouba[1]*, Jan Tíkal[1], Petr Císař[1], Lukáš Veselý[1], Martin Fořt[1], Josef Příborský[1], Jiří Patoka[2], Miloš Buřič[1]

[1] University of South Bohemia in České Budějovice, Faculty of Fisheries and Protection of Waters, South Bohemian Research Center of Aquaculture and Biodiversity of Hydrocenoses, Zátiší 728/II, CZ-389 25 Vodňany, Czech Republic

[2] Department of Zoology and Fisheries, Faculty of Agrobiology, Food and Natural Resources, Czech University of Life Sciences Prague, Kamýcká 129, CZ-165 21 Prague 6 - Suchdol, Czech Republic

***Correspondence:** Antonín Kouba, akouba@frov.jcu.cz, tel.: +420 389 034, fax: +420 389 034 745

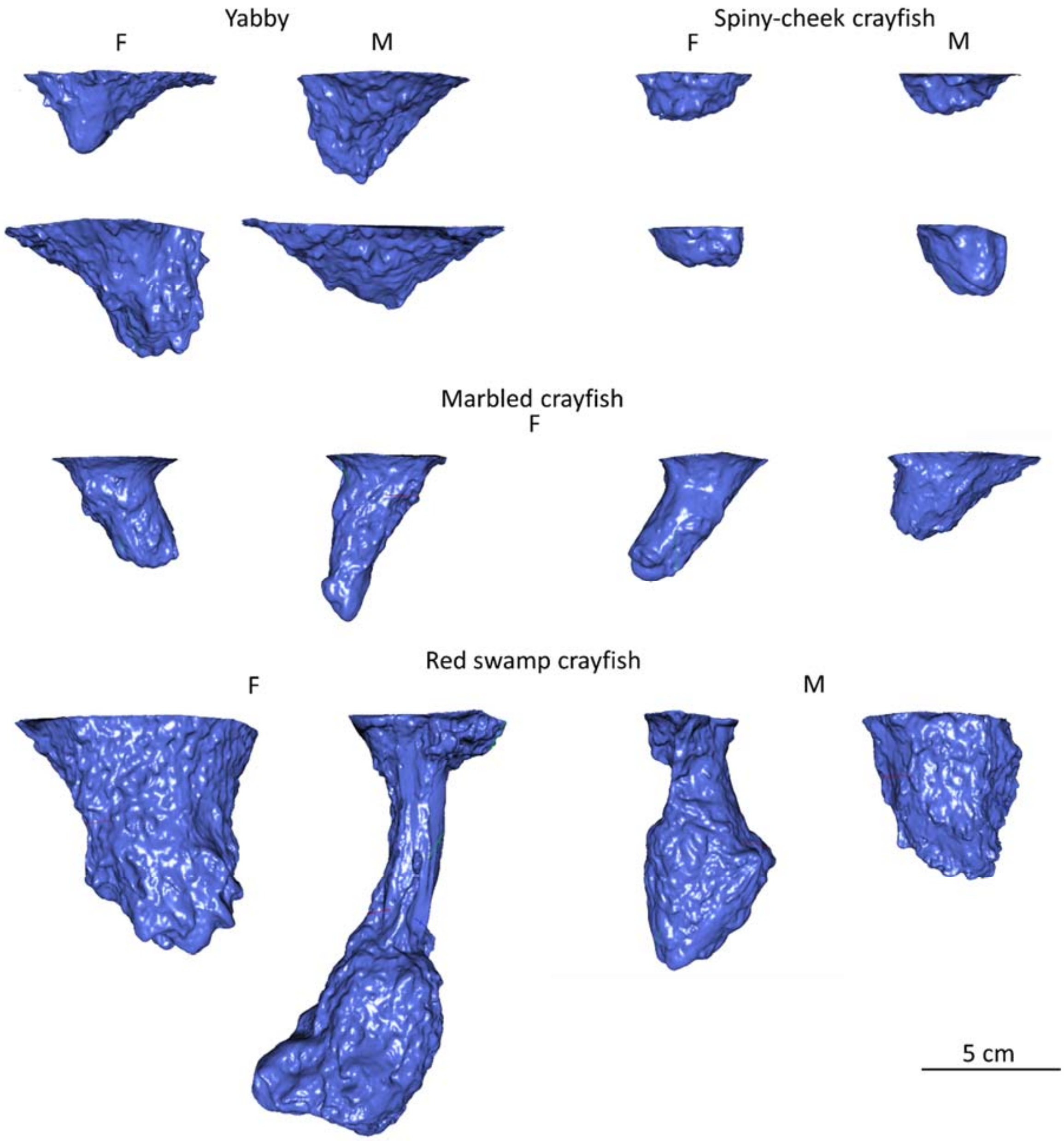

Figure S1. Further examples of burrows constructed by crayfish species involved in the experiment.

Table S1. Size distribution of sand and clay particles evaluated in the analytical laboratory of AGRO-LA Inc. (Jindřichův Hradec, Czech Republic) and provided by the supplier, respectively.

**Sand**		**Clay**	
**Size class (mm)**	**Portion (%)**	**Size class (µm)**	**Portion (%)**
< 0.4	16.6	< 2	48.2
0.4 – 0.5	7.8	2 – 4	9.0
0.5 – 0.63	5.5	4 – 10	11.6
0.63 – 0.8	9.6	10 – 20	9.0
0.8 – 1.0	32.5	20 – 40	7.4
1.0 – 1.25	12.2	40 – 63	3.2
1.25 – 1.4	2.3	> 63	11.6
1.4 – 2.0	8.2		
> 2.0	5.4		

Table S2. Biometry of crayfsh involved in the experiment. Data are presented as mean±SD and range.

**Species**	**n**	**Capapace lenght (mm)**	**Weight (g)**
Astacus astacus	4	51.8±5.0 (43.7–57.3)	37.7±11.2 (19.0–48.9)
Astacus leptodactylus	4	51.2±8.9 (41.8–63.1)	42.0±23.2 (18.6– 6.3)
Austropotamobius torrentium	4	31.4±2.2 (29.1–33.8)	10.2±3.1 (6.9–13.3)
Cherax destructor	14	37.5±3.9 (26.9–43.3)	20.0±6.0 (6.3–29.1)
Orconectes limosus	10	29.9±3.3 (22.2–33.7)	7.9±2.1 (4.5–11.3)
Pacifastacus leniusculus	10	46.5±7.1 (26.2–54.9)	34.6±4.7 (25.8–42.9)
Procambarus clarkii	24	37.7±2.7 (32.3–44.1)	14.3±3.1 (7.9–21.9)
Procambarus fallax* f. *virginalis	12	26.9±4.4 (22.1–35.4)	6.0±3.0 (3.2–11.7)
